# Lessons Learned from the Pandemic in the UAE: Children COVID-19 Vaccine Hesitancy and Its Impact on the Choice of Distance versus Face-to-Face Learning Modalities: An In-Depth Analysis of a National Study

**DOI:** 10.3390/vaccines11101598

**Published:** 2023-10-16

**Authors:** Zelal Kharaba, Yassen Alfoteih, Karem H. Alzoubi, Sayer Al-Azzam, Ahmad Al-Azayzih, Hala J. Al-Obaidi, Ahmed Bahaaeldin Awad, Yahya H. Dallal Bashi, Rahaf Ahmed, Alaa M. Khalil, Raneem Al Ahmad, Mamoon A. Aldeyab, Feras Jirjees

**Affiliations:** 1 Department of Clinical Pharmacy, College of Pharmacy, Al Ain University, Abu Dhabi Campus, Abu Dhabi 112612, United Arab Emirates; 2 Faculty of Medical Sciences, Newcastle University, Newcastle upon Tyne NE2 4HH, UK; 3 Department of Dental Surgery, City University Ajman, Ajman 18484, United Arab Emirates; a.yassen@cu.ac.ae; 4 Department of General Education, City University Ajman, Ajman 18484, United Arab Emirates; 5 Department of Pharmacy Practice and Pharmacotherapeutics, College of Pharmacy, University of Sharjah, Sharjah 27272, United Arab Emirates; khalzoubi@just.edu.jo (K.H.A.); fjirjees@sharjah.ac.ae (F.J.); 6 Department of Clinical Pharmacy, Faculty of Pharmacy, Jordan University of Science and Technology, Irbid 22110, Jordan; salazzam@just.edu.jo (S.A.-A.); aaazayzih@just.edu.jo (A.A.-A.); 7 School of Pharmacy, Queen’s University Belfast, Belfast BT9 7BL, UK; halobaidi01@qub.ac.uk (H.J.A.-O.); ydallalbashi01@qub.ac.uk (Y.H.D.B.); 8 Department of Clinical Pharmacy, Burjeel Medical City Hospital, Abu-Dhabi 7400, United Arab Emirates; aawad3355@gmail.com; 9 Abbott LaboratoriesAlphamed Company Limited, Abu Dhabi 4236, United Arab Emirates; rahafalyrahafaly@gmail.com; 10 Al Thiqa Pharmacy Group, Abu Dhabi 47612, United Arab Emirates; alaakhalil20001@gmail.com; 11 Pharmacy Intern, Cleveland Clinic Abu Dhabi, Abu Dhabi 112412, United Arab Emirates; raneem.alahmad@gmail.com; 12 Department of Pharmacy, School of Applied Sciences, University of Huddersfield, Huddersfield HD1 3DH, UK; m.aldeyab@hud.ac.uk

**Keywords:** COVID-19, children vaccination, vaccine hesitancy, parents’ perception, distance learning, national study

## Abstract

This study addresses the crucial aspect of childhood COVID-19 vaccination and its impact on parental decisions concerning learning modalities during the pandemic. This study aimed to gauge parental hesitancy towards vaccinating their children and its influence on choosing between distance and face-to-face learning options. Following STROBE guidelines for cross-sectional studies, this study surveyed 1973 parents in the United Arab Emirates using Google Forms during the COVID-19 pandemic. The results revealed that while more than half of the parents (51.6%) were willing to vaccinate their children if the COVID-19 vaccine was accessible and affordable, a significant majority (91.2%) expressed concerns about the rapid vaccine development process, which was the primary reason for vaccine rejection. Interestingly, a sizable portion (55.3%) had experienced online learning in the previous academic term, and, of those, 59.6% believed it negatively influenced their children’s academic performance. Consequently, 66.4% expressed intent to shift their children back to face-to-face learning once feasible. Significantly, parents with medical backgrounds were more inclined (91.6%) to opt for face-to-face schooling compared to those without such backgrounds. Logistic regression analysis indicated associations between sociodemographic characteristics, educational level and background, and the decision to return children to face-to-face learning. Interestingly, when it comes to vaccine hesitancy, a noteworthy connection exists between the parents’ reluctance to vaccinate their children and their preference for distance learning. In fact, parents who responded negatively to vaccinating their children against COVID-19, if the vaccine was available, showed a clear preference for the distance learning modality (*p*-value < 0.0001). This study underscores the complex interplay of factors and community perspectives shaping parental acceptance of childhood COVID-19 vaccination. The development pace of vaccines significantly influences parents’ attitudes and beliefs about vaccination programs. Parents’ medical backgrounds exhibit a clear correlation with their perceptions of sending children back to school safely. This highlights the potential impact of parental medical knowledge on decision making, emphasizing the need to consider parents’ professional backgrounds when devising education- and vaccination-related policies.

## 1. Introduction

Child vaccination against COVID-19 is vital in controlling the spread of the virus and ensuring the safety of children in educational settings. The United Arab Emirates (UAE) has taken proactive measures by implementing preventive strategies and launching a national vaccination program [1]. However, parental resistance and hesitancy towards vaccinating their children due to safety and efficacy concerns pose challenges. In order to control the spread of the coronavirus and safeguard children in educational environments, the use of the COVID-19 vaccine was authorized as a means of protecting children from infection. In fact, the COVID-19 vaccine does not only protect children in educational settings but also plays a crucial role in shielding other family members from potential transmission when any individual within these groups becomes infected. Moreover, administering the vaccine effectively reduces the severity of symptoms among the family members [2].

Rosenstrom et al. 2022 published a recovered simulation model study, and concluded that high vaccination uptake among both children and adults is necessary to mitigate the increase in infections from mask removal in schools and workplaces [3]. Indeed, child vaccination against COVID-19 plays a significant role in safeguarding children’s health and minimizing the risk of transmission in educational settings. The UAE’s national vaccination program reflects the government’s commitment to ensuring the well-being of its citizens, particularly that of the younger population [1]. According to the World Health Organization (WHO), child vaccination programs are crucial for reducing the burden of infectious diseases and achieving herd immunity [2]. By vaccinating children, a protective shield can be created, allowing educational institutions to function with reduced health risks [4].

Despite the UAE’s efforts, some parents exhibit resistance and hesitancy towards vaccinating their children against COVID-19. Concerns regarding vaccine safety and efficacy contribute to parental hesitancy [5]. It is essential to explore parents’ acceptance, perception, and hesitancy towards COVID-19 vaccination to address their concerns effectively and promote informed decision making. Several articles have discussed the impact of students’ vaccination status on the choice of distance learning versus face-to-face delivery methods [6,7,8,9]. However, most of these studies focused on college students and there is still a lack of studies that discuss this impact on school children. Vaccination status may influence parents’ decisions regarding the learning environment for their children as vaccinated children may have a higher likelihood of participating in face-to-face learning, considering the reduced health risks associated with immunization [10]. In fact, the UAE ministry of education across all schools in the UAE announced that distance learning continued until the end of the academic year 2020–2021. After that, all parents across all regions of the UAE (before, during and after the completion of this study) had the choice between distance and face-to-face learning. In contrast, the parents of unvaccinated children might opt for distance learning due to concerns about exposure to the virus. Therefore, vaccination can play a role in shaping educational decisions, affecting the balance between distance and face-to-face learning. The question of whether vaccine hesitancy might shape educational decisions is worth investigation. Communication campaigns focusing on vaccine safety, efficacy, and long-term benefits can help address parental concerns [11]. By fostering trust and providing accurate information, health authorities can alleviate vaccine hesitancy and encourage parents to make informed decisions regarding their children’s immunization [12].

This study aimed to identify the level of parental hesitancy to vaccinate their children against COVID-19 and its impact on parents’ decisions of distance vs. face-to-face learning modalities for their children.

The findings of this study can assist health authorities and policy makers in comprehending the factors contributing to parental hesitancy in vaccinating their children. With this understanding, authorities can develop effective strategies to educate parents about the advantages of vaccination not only for the COVID-19 vaccine but also for other vaccines targeting children, such as the influenza vaccine and the human papillomavirus, HPV, vaccine. These efforts are particularly crucial in regions where the utilization of these vaccines remains relatively low [13,14,15]. By promoting awareness and addressing concerns, health authorities can enhance vaccination rates and improve public health outcomes in the country and the wider region.

## 2. Materials and Methods

### 2.1. Design of the Study

Strengthening the Reporting of Observational Studies in Epidemiology reporting guidelines for cross-sectional studies, STROBE, was adopted and followed as a protocol for conducting this study.

For data collection, this study employed a descriptive questionnaire-based cross-sectional online survey conducted within the United Arab Emirates (UAE). The survey targeted parents aged 18 years and above who voluntarily provided their informed consent to participate. Eligibility criteria stipulated that participants must have been residents of the UAE during the COVID-19 pandemic and have children enrolled in primary or secondary schools. Individuals without children were excluded from the study.

The recruitment of participants was executed through diverse social media platforms, and the survey questionnaire was made available in both English and Arabic to cater to the linguistic diversity of parents in the UAE. The distribution of the questionnaire was facilitated through the utilization of Google Form^®^ to individuals with online accessibility. The survey link, accompanied by an informed consent form, was disseminated through widely used platforms such as WhatsApp^®^, Instagram^®^, Facebook^®^, Twitter^®^, and email. This dissemination phase transpired over a three-month duration, encompassing the period from 1 June to 1 September 2021. The participants were encouraged to share the survey with their acquaintances, family members, and social networks. Comprehensive explanations regarding confidentiality and anonymity policies were provided and clearly communicated to all respondents.

### 2.2. Ethical Approval

This study received ethical permission from the research ethics committee of Al-Ain University, Abu Dhabi, United Arab Emirates, (AAU-REC-B3, February 2021).

### 2.3. Sample Size

The recruitment of participants was conducted through convenience sampling, with the objective of achieving a representative sample size of at least 386, as determined by both prior research and the Raosoft sample size calculator [16]. The selected sample size was established with consideration of a 95% confidence level, and a margin of error of 5%. Nevertheless, in pursuit of a more comprehensive and inclusive representation, the researchers made the deliberate choice to expand the final sample size to 1973 participants for inclusion in the study.

### 2.4. Research Instrument

A comprehensive questionnaire was constructed following a thorough review of the existing literature [1,7,8,10,17,18]. The survey instrument was initially developed in English and subsequently underwent translation into Arabic by two proficient bilingual experts. A range of domains was encompassed in the semi-structured questionnaire, including sociodemographic characteristics, lifestyle factors, and health-related awareness inquiries pertaining to the COVID-19 pandemic. The questionnaire systematically evaluated the participants’ inclination towards accepting the COVID-19 vaccine and their readiness to extend it to their children. Additionally, the survey explored the potential association between the chosen educational modality (namely, distance or face-to-face learning) and children’s vaccination status, thereby shedding light on any potential interdependencies. Furthermore, the questionnaire delved into the sources of information that parents accessed with regard to the COVID-19 pandemic and the corresponding vaccine.

The inaugural segment of the questionnaire gathered sociodemographic information from the respondents, encompassing details such as age, sex, residential location, education level, academic discipline, and employment situation. The subsequent segment of the questionnaire inquired into parents’ viewpoints pertaining to the COVID-19 vaccine, encompassing aspects such as their awareness, reception, and reservations concerning the COVID-19 vaccine both in a general context and specifically concerning children. The concluding section of the questionnaire directed its focus toward parents’ determinations concerning the re-enrollment of their children in educational institutions and their apprehensions regarding the safety of vaccines.

An evaluation was conducted employing a five-point Likert scale to appraise parents’ inclinations regarding the resumption of their children’s attendance at educational institutions and their apprehensions regarding vaccine side effects, aiming to determine parents’ preference for learning modalities for their children, allowing them to choose between distance or face-to-face learning, and thus establishing a relationship between children’s COVID-19 vaccination and the choice of learning mode. The five-point Likert scale included the following options: strongly disagree, disagree, neutral, agree, strongly agree. The strongly disagree and disagree scales were combined into one category, “No”; the neutral responses stayed in the same category; and the agree and strongly agree scales were combined into the “Yes” category in the table. Furthermore, the investigation encompassed a series of Yes/No inquiries aimed at capturing parental perspectives encompassing their knowledge, acceptance, and reservations pertaining to the COVID-19 vaccine in a broader context, as well as its specific relevance to children.

### 2.5. Validation and Reliability of the Study Questionnaire

The principal investigator orchestrated a virtual assembly in which four academic scholars specializing in health and medical sciences within the UAE, within four non-medical individuals, were extended invitations to validate the questionnaire’s content. Each participant was tasked with appraising every individual item within the questionnaire utilizing a scale that ranged from 1 to 10.

The evaluation criteria encompassed aspects such as lucidity, pertinence, appropriateness, question length, and the temporal requirement for completing the questionnaire.

The computed average ratings for attributes such as clarity, relevance, appropriateness, question length, and time consumption yielded scores of 9.24 (SD ± 1.67), 9.84 (SD ± 2.09), 8.77 (SD ± 1.14), 9.24 (SD ± 1.67), and 8.53 (SD ± 2.67), respectively. Subsequently, the observations, insights, and suggestions proffered by the participants were meticulously considered and integrated into the questionnaire. Consequently, the questionnaire underwent necessary modifications and refinements guided by the feedback received from these experts.

Subsequently, the research team executed a preliminary trial utilizing the verified rendition of the questionnaire to evaluate its reliability and comprehensibility. The pilot test entailed 25 individuals who were tasked with the completion of the survey.

Their role extended to the provision of feedback concerning any queries or phrasings that might have the potential to hinder their understanding of the questionnaire’s content. In the ensuing phase, the accumulated responses underwent analysis through the utilization of Statistical Package for the Social Sciences (SPSS) version 26 (IBM Corp, Armonk, NY, USA). This analysis was conducted to ascertain the internal consistency of the questionnaire’s constituent items.

### 2.6. Statistical Analysis

The collected data underwent meticulous organization within an Excel spreadsheet and was then subsequently imported into the Statistical Package for the Social Sciences (SPSS) software version 26 for thorough analysis. Descriptive statistics were employed to summarize the patients’ characteristics, encompassing frequencies, mean values, and standard deviations for categorical and continuous variables, respectively. Univariate associations were investigated using appropriate statistical tests like chi-square for categorical variables to identify associations between sociodemographic characteristics and parents’ perception regarding learning methods. Logistic regression analysis was conducted to examine the parents’ plans to return their children to face-to-face learning.

The explanatory variables included age, sex, educational level and background, residence area. The results were presented as odds ratios (OR). All reported *p*-values underwent two-sided evaluation, with significance determined at the 0.05 level.

Multivariate analysis was performed to assess the relationship between various independent variables and the parents’ plans to return their children to face-to-face learning. The results were presented as odds ratios (OR). All reported *p*-values were two-sided, with a significance level set at 0.05.

## 3. Results

### 3.1. Sociodemographic Characteristics

Table 1 shows the characteristics of the study participants in addition to the safety practices observed for children during the pandemic. This study involved a total of 1973 participants. Among the participants, the majority were females, accounting for 87.3% of the total. Over half of the participants (59.3%) fell within the age range of 18 to 40 years old and possessed a bachelor’s degree (50.7%). Furthermore, a large proportion of the participants (69.8%) came from non-medical backgrounds. Additionally, more than half of the participants (50.5%) reported residing with elderly individuals aged 65 years or over. In terms of safety and protective measures to prevent COVID-19 infection among children, the parents reported that more than half of the children (54.8%) had received a flu vaccine. Furthermore, a significant majority of the children (77.9%) regularly used masks in public settings. Additionally, when feeling unwell, the majority of children (71.3%) stayed at home rather than attending school or other public places. These findings indicate a positive adherence to safety practices among the children surveyed.

### 3.2. Parents’ Perception of the COVID-19 Vaccine, and Vaccination’s Effect on Learning

According to Table 2, over half of the parents (51.6%) expressed their willingness to vaccinate their children aged 5 years and above if the COVID-19 vaccine was accessible and affordable. When parents were questioned about reasons for not vaccinating their children if the COVID-19 vaccine is accessible and affordable, the majority of parents (91.2%) reported concerns about the speed of vaccine development as the primary reason for rejecting vaccination. Additionally, a significant number of parents (87.8%) relied on social media as their main source of information regarding COVID-19 vaccination for children.

In terms of the learning modality used by the children, more than half of them (55.3%) attended their classes online during the last academic term (the second academic semester—from March to the end of June 2021). Additionally, a considerable proportion of parents (59.6%) acknowledged that distance learning had an impact on their children’s academic performance. As a result, approximately two-thirds of the participants (66.4%) expressed their intention to transition their children back to face-to-face learning once it becomes feasible. These findings highlight the preferences and considerations of parents regarding their children’s learning methods.

Many participants (61.5%) believed that vaccinating children would facilitate the resumption of face-to-face education in schools, although less than half of the participants (46.3%) believed that the vaccination of children was primarily aimed to protect them. Nevertheless, around two-thirds of the participants (63.5%) expressed general concerns and worries regarding the ongoing COVID-19 pandemic. These findings reflect the mixed beliefs and concerns among the participants regarding the role of child vaccination and the overall impact of the pandemic.

### 3.3. Educational Background

Table 3 demonstrates significant differences (*p* < 0.0001) in parents’ perspectives based on their educational background and the learning methods. Parents with a medical background showed a higher inclination (91.6%) to return their children to face-to-face learning compared to parents with a non-medical background (55.5%). Additionally, a lower percentage of parents with a medical background (24.9%) expressed concerns about the safety of their children attending school physically, in contrast to a higher ratio of parents with a non-medical background (59.3%). However, a higher percentage of parents with a medical background (79.0%) had general concerns about the COVID-19 pandemic compared to the other group (56.8%). Significant differences were also observed in the perception of the COVID-19 vaccine’s role in helping children return to schools and protecting them from infection (*p* < 0.0001). A high percentage of parents with a medical background (83.2%) believed in the vaccine’s ability to assist with school re-entry, and a high percentage of parents (72.0%) believed in the vaccine’s protective benefits compared to a lower percentage in the non-medical background group, with values of 52.1% and 35.2%, respectively.

### 3.4. Association between Sociodemographic Characteristics and Parents’ Perception of Distance Learning

Table 4 shows the association between the sociodemographic characteristics of the participants and parents’ perception of the three learning methods: distance, face-to-face, and hybrid. The analysis reveals a statistically significant association between age and parents’ preference for learning methods (*p* < 0.0001). There was more of a preference towards the distance learning modality, with values of 71.4% of older parents (over 51 years of age). For the residence area, the results indicated a statistically significant association with parents’ preference for learning methods (*p* < 0.0001). Generally, the choice of the distance learning modality was the highest in the three residence areas—54.2% in Abu Dhabi, 44.4% in Dubai, and 60.4% in the Northern Emirates (that includes five Emirates)—compared to the other learning modalities (face-to-face and hybrid method). Furthermore, the results revealed a statistically significant association (*p* < 0.0001) between parents’ educational level and their perception of learning modalities. Parents with a high school education displayed a strong preference for distance learning. In contrast, parents with different higher educational levels showed varying degrees of preference for distance learning: 38.8% of undergraduate students, 55.2% of parents with a bachelor’s degree, 66.7% of parents with a master’s degree, and 55.9% of parents with a PhD degree. These findings highlight the influence of educational level on parents’ preferences for different learning methods.

Finally, the results showed a significant association (*p* < 0.0001) between parents’ educational background (medical or non-medical) and their preferred learning method. Among parents with a medical background, 34.9% preferred distance learning, 29.7% preferred face-to-face learning, and 35.4% preferred hybrid learning. In contrast, among parents with a non-medical background, a higher percentage (64.1%) preferred distance learning, while 17.4% preferred face-to-face learning, and 18.5% preferred hybrid learning. When it comes to vaccine hesitancy, a noteworthy connection exists between parents’ reluctance to vaccinate their children and their preference for distance learning. In fact, parents who responded negatively to vaccinating their children against COVID-19, if the vaccine was available, showed a clear preference for the distance learning modality (*p*-value < 0.0001).

### 3.5. Multivariate Analysis of Returning the Children to Face-to-Face Learning

According to the logistic regression analysis presented in Table 5, several sociodemographic characteristics of the participants showed significant associations with parents’ plans to return their child/children to face-to-face learning. Specifically, among two groups of parents younger than 40 years (*p* value 0.009 and <0.0001, respectively), different educational backgrounds (non-medical vs. medical background) (OR = 0.075) were found to be significant factors. Regarding hesitancy to vaccinate, parents who reported No to vaccinate their children were less likely to send their children to school (OR = 0.423, *p*-value 0.000). These results suggest that these variables played a role in the parents’ decision-making process regarding the return to face-to-face learning for their children.

## 4. Discussion

Ensuring the safety of children in educational settings and controlling the spread of the virus are crucial factors that make child vaccination against COVID-19 imperative. The UAE’s proactive approach to implementing preventive measures and launching a national vaccination program is commendable [1]. However, the COVID-19 pandemic and the subsequent implementation of lockdowns have had a profound impact on various aspects of life. The UAE government mandates a series of vaccinations for children from birth until they reach Grade 11. These vaccinations are designed to prevent a range of diseases, including BCG, Bacillus Calmette–Guérin (for tuberculosis); DPT, diphtheria, pertussis, and tetanus; DTaP, diphtheria, tetanus, and acellular pertussis; Hep B, hepatitis B; and many others. As of May 2021, the Ministry of Health and Prevention has approved the emergency local use of vaccines for children between 12 and 15 years of age after clinical trials and assessment. However, and to the best of our knowledge, there is a lack of data describing the overall percentage of children vaccination in the UAE (to date).

One of the important results of the pandemic was the disruption of traditional education systems, which led to the shift towards distance learning. The current study aimed to assess the importance of children vaccination against COVID-19 infection and the choice of learning method. Furthermore, it highlighted the need to address vaccine hesitancy among the parents and its potential impact on educational decisions. Around 2000 parents in the UAE participated in this study, and these participants had different ages and educational backgrounds. This study was conducted during the COVID-19 pandemic (June to September 2021) in retrospect when online teaching was the main learning method in the UAE.

One finding of the study was that a high percentage of children followed the safety instructions during the pandemic, such as wearing masks, and isolating at home when they felt unwell. This finding reflects the persistent efforts of the UAE government in educating and increasing the public’s awareness of the preventive measures against COVID-19 infection [19], which have positively affected the parents’ belief in wearing face masks and children vaccination.

Vaccine hesitancy among parents was notable, with a high preference for physically returning their children back to school.

In a compelling discovery within our study, a notable association emerged between parental hesitancy to vaccinate their children and the selection of distance learning as an educational method. Specifically, parents who expressed reservations about the availability of a COVID-19 vaccine tended to favor the distance learning approach, while those who displayed confidence in vaccine availability preferred face-to-face learning (*p*-value < 0.0001). Numerous scholarly works have delved into student perceptions of virtual versus in-person teaching during the COVID-19 pandemic (references [9,10]), while another body of literature has explored parental attitudes towards vaccinating their children against COVID-19 (references [6,7]). Surprisingly, there exists a research gap in the examination of the interconnections between these factors, underscoring the significance of our current study.

This study explored various factors that contributed to parents’ decisions regarding returning their children to school, including non-medical educational background and concerns about vaccine development. A study by Aedh et al., 2022, reported that 72.2% of parents showed vaccine hesitancy and were 9.5 times less likely to immunize their children against COVID-19, while 27.8% of parents were ready to vaccinate their children against the virus as soon as possible, compared to 15.51% of parents who were not at all interested in vaccinating their children. In addition, the lack of adequate safety data, potential future consequences, and vaccine efficacy were the main concerns regarding COVID-19 vaccines [20]. The findings of another study conducted in the UAE revealed that the public is not sufficiently informed about the efficacy or side effects of the COVID-19 vaccine [21]; thus, increasing the public’s awareness will help parents make informed decisions regarding vaccinating their children and potentially increase vaccine acceptance. Furthermore, a study by Lazarus et al. 2022 reported that parental willingness to vaccinate their children in many countries slightly increased by around 2% between 2021 and 2022 [22].

It is worth noting that a large proportion of parents in the current study relied on social media as the main source of information related to vaccinating children against COVID-19. This confirms the impact of social media platforms in shaping parents’ perceptions of vaccination. Generally, there is confusion regarding the accuracy and reliability of information shared through various channels to address misinformation and promote informed decision making related to vaccine [23]. In addition, studies that have evaluated social media content related to COVID-19 vaccination indicated low perceived benefits of vaccination, and a lack of information [24,25]. This was evident with parents who refused to vaccinate their children, which was the case for around half of the participants in this study. Many published studies have indicated that there was low trust in vaccine development among people [26,27,28,29,30]. However, these findings may vary across regions and populations. These studies indicated that parental concerns about the speed of vaccine development were prevalent. The perceived rapid timeline raised doubts among some parents regarding the long-term safety and efficacy of the vaccines.

The impact of distance learning on academic performance and the desire to return to face-to-face learning shed light on the need for applicable strategies to ensure quality education during the pandemic. The findings also highlighted the perceived association between children’s vaccination and the resumption to face-to-face learning. Many participants believed that vaccination would make it easier for their children to physically return to school. However, less than half of participants believed that vaccinating children is primarily intended to protect them. The mixed beliefs and concerns among parents regarding children’s vaccinations and the overall impact of the pandemic reflect the complexity of addressing public perceptions and ensuring the wellbeing of children and families. There was a similarity between these findings and those of some studies among Arab communities [31].

Regarding the impact of the pandemic on the preferred learning method, this study indicated a trend among parents towards supporting their children’s return to face-to-face learning as early as possible. This indicates a preference for in-person schooling among participants, likely due to the perceived benefits of face-to-face interaction, socialization, and better learning experiences in a physical classroom. This indicates less concern for parents regarding the distance learning method, as indicated by their answer that distance learning affected their children’s academic performance, which revealed that parents are aware of the challenges associated with distance education and its potential impact on learning outcomes. Several studies examined the experiences of students during the transition to distance learning amid the pandemic [32,33,34] and many of them found that a considerable number of participants preferred face-to-face learning due to factors such as declines in children’s learning motivation and cognitive abilities. Students faced challenges in distance learning such as a lack of engagement, difficulty in understanding complex topics, and a decrease in academic performance compared to face-to-face learning. On the other hand, the findings of other studies revealed that students appreciated the convenience and accessibility of distance education, although the learning experiences were significantly different across school years and reflected challenges and differences according to the disciplinary area. Indeed, it is clear that most studies revealed that students appreciated the convenience and accessibility of distance education amid the pandemic. On the other hand, the preferences of parents and students regarding distance and face-to-face learning during the COVID-19 pandemic was discussed in several studies [35,36,37]. In addition, the impact of the sudden shift to distance learning on students’ academic performance was discussed in the literature [38,39,40]. The findings suggest that distance learning posed challenges, including technical difficulties, reduced interaction, and decreased motivation, which negatively affected academic scores.

There were several significant associations observed in the study, providing insights into the influence of sociodemographic factors such as age, residence area and educational level of parents, on their preferences for distance learning during the COVID-19 pandemic. The effect of sociodemographic factors was varied among studies in the field. A study conducted in Nigeria indicated that other factors have an effect on parents’ preferences for distance learning during the pandemic, such as the sex of the parents, and the economic and financial situation of the participants [41].

The findings of this study also indicated several significant associations that highlighted the differences between the medical and non-medical backgrounds of the parents and their choice of distance vs. face-to-face learning and the COVID-19 vaccine. The educational background of parents who participated in this study (medical vs. non-medical backgrounds) was clearly associated with their perception of the safety of their children returning to school. The data reveal that a significantly higher proportion of parents with a medical educational background planned to return their children to face-to-face learning, expressing feelings that their children are not in danger, compared to parents with a non-medical background. According to this finding, we assumed that parents with medical backgrounds believed that children vaccination would help schools return to face-to-face teaching and in the protection effect of vaccine for their children more than non-medical parents. The parents with a non-medical background had a greater preference for the distance learning option and a lower preference for sending their children to school physically. This is most possibly due to concerns about the potential health risks their children might face when attending face-to-face classes during the COVID-19 pandemic. Additionally, parents without a medical background revealed weaker belief in the notion that vaccinating their children would facilitate the return to face-to-face education as they have a weaker belief that the COVID-19 vaccine would effectively protect their children and are less convinced about the role of vaccination in creating a safer environment for in-person learning. In addition, these parents had more uncertainties regarding the vaccine’s ability to provide adequate protection against the virus for their children. On the other hand, parents without a medical background displayed less concern about the COVID-19 pandemic at the time of the study, which may reveal they had a comparatively lower level of awareness regarding the pandemic. Several studies explored parents’ willingness to send their children back to school after the COVID-19 outbreak [42,43,44] and many of them explored parental attitudes and concerns regarding the return to face-to-face learning during the COVID-19 pandemic. It has been found that parents from various fields expressed concerns about the health and safety of their children in physical school settings, leading to hesitancy regarding the return to face-to-face learning.

Most of the studies showed that parents were more cautious about vaccinating their children than vaccinating themselves [45,46,47,48,49]. Moreover, due to the different medical system backgrounds and composition of studies in different countries, there is great heterogeneity among the willingness rates, making direct comparisons difficult [48]. Differences among respondents across different regions reflected different COVID-19 policies and cultural backgrounds. In our study, parents from non-medical fields often expressed higher levels of concern and hesitancy about the return to physical school due to fears of potential COVID-19 transmission. It is important to consider that parental attitudes and intentions regarding the return to face-to-face learning can vary based on individual circumstances, regional context, and evolving public health guidelines.

### Strengths and Limitations of the Study

Our study has a few limitations. As this study used electronic surveys and a convenience sampling technique, this may limit the generalizability of the results. Additionally, there is a possibility of selection bias due to the nature of the online surveys. Furthermore, this study relied on self-reported data, which can be subjected to recall bias. Participants may have difficulty accurately remembering past events or experiences, leading to potential inaccuracies in the information collected. However, and to the best of our knowledge, this is the first national study with a representative sample size across all Emirates in the UAE to investigate parents’ vaccine hesitancy and its impact on the choice of distance versus face-to-face learning modalities. The results of this study have the potential to provide valuable insights for health authorities and policy makers in comprehending the underlying factors that contribute to parental hesitancy when it comes to vaccinating their children. By gaining a deeper understanding of these factors, authorities can formulate effective strategies aimed at educating parents about the numerous benefits associated with vaccination. These efforts hold particular significance in regions where vaccine utilization remains relatively low. By fostering awareness and addressing the concerns of parents, health authorities have the potential to significantly enhance vaccination rates and consequently improve public health outcomes not only within the country but also across the broader region.

## 5. Conclusions

The acknowledged effectiveness of COVID-19 vaccines against the virus contrasts with the intricate challenge of gaining public acceptance, especially among parents regarding children’s vaccination. This complexity is derived from various factors and community viewpoints. Concerns about the vaccines’ long-term effects on children, coupled with rapid development, significantly impact parental attitudes toward vaccination programs. Hesitancy and inadequate information impede children’s COVID-19 vaccine uptake.

Our study further revealed a compelling finding: parents who expressed reservations about the availability of a COVID-19 vaccine tended to favor the distance learning approach, while those who displayed confidence in vaccine availability preferred face-to-face learning. This underscores the importance of understanding how vaccination beliefs intersect with educational preferences. Prioritizing evidence-based communication and education is vital to provide parents with accurate resources for informed decisions. Equipping healthcare professionals to address parents’ concerns is equally important. Policy makers should ensure access to precise vaccination information. Notably, parents’ educational backgrounds influence their perceptions of safely sending children back to school. These multifaceted parental beliefs warrant targeted strategies to be carried out by educators and healthcare providers to enhance student safety and informed decision making.

## Figures and Tables

**Table 1:** Participants’ characteristics and safety practices among children (*n* = 1973).

Variables		N (%)
Sex	Male	251 (12.7%)
	Female	1722 (87.3%)
Age categories	18–30 years	599 (30.4%)
	31–40 years	579 (29.3%)
	41–50 years	571 (28.9%)
	≥51 years	224 (11.4%)
Residence area	Abu Dhabi	863 (43.7%)
	Dubai	295 (15.0%)
	Northern Emirates *	815 (41.3%)
Educational level	High school	12 (0.6%)
	Undergraduate students	317 (16.1%)
	Bachelor	992 (50.3%)
	Postgraduate (master’s degree)	401 (20.3%)
	Postgraduate (PhD)	247 (12.5%)
Educational background	Medical	596 (30.2%)
	Non-medical	1377 (69.8%)
Living with an elderly person (65 years old and over)	Yes	996 (50.5%)
The children received flu vaccine	Yes	1081 (54.8%)
The children used a mask in public	Yes	1537 (77.9%)
The children stayed at home when they feel slightly unwell	Yes	1407 (71.3%)

* Northern Emirates include Sharjah, Ajman, Um Al Quwain, Ras Al Khaimah, and Fujairah.

**Table 2:** Parents’ perspectives on the COVID-19 vaccine and its impact on learning.

Questions	Collected Answers	N (%)
If the COVID-19 vaccine is available and affordable for children over the age of five, would you give it to your child?	No	953 (48.4%)
	Yes	1020 (51.6%)
If your answer is “No”, how best describes the reason why?	I am concerned about side effects and safety	84 (8.8%)
	I am concerned about the speed that the vaccine was made	869 (91.2%)
Have you or someone you know gotten COVID-19 infection?	No	792 (40.1%)
	Yes	1181 (59.9%)
Have any of your children contacted with a COVID-19 patient?	No	1134 (57.5%)
	Yes	839 (42.5%)
What is your main source of information about the COVID-19 vaccination for children?	Social media	1732 (87.8%)
	TV	65 (3.3%)
	Official Health authority and WHO	176 (8.9%)
Did your child/children attend the school face-to-face, distance, or hybrid education during the last academic term?	Distance learning	1091 (55.3%)
	Face-to-face learning	416 (21.1%)
	Hybrid learning	466 (23.6%)
For parents selected Online/hybrid learning: Did distance (online) learning affect you children academic scores?	No	630 (40.5%)
	Yes	927 (59.5%)
For parents selected Online/hybrid learning: Do you plan to return your child/children back to face-to-face learning if this is possible?	No	523 (33.6%)
	Yes	1034 (66.4%)
Do you believe that children vaccination will help schools to return to face-to-face education?	No	759 (38.5%)
	Yes	1214 (61.5%)
Do you believe that children vaccination will protect the children more?	No	586 (29.7%)
	Yes	914 (46.3%)
	Not sure	473 (24.0%)
After more than one year of the pandemic, are you concerned and/or worried about the pandemic?	No	720 (36.5%)
	Yes	1253 (63.5%)

**Table 3:** Association between parents’ educational background and awareness toward distant learning, vaccination effect on learning and importance of the COVID-19 vaccine.

Questions		Educational Background		*p* Value
		Medical (*n* = 596)	Non-Medical (*n* = 1377)	
	*n* (%)			
Parents’ perception of the safety of their children’s return to school				
Do you plan to return your child/children back to face-to-face learning when it is possible?	No	50 (8.4%)	613 (44.5%)	<0.0001 *
	Yes	546 (91.6%)	764 (55.5%)	
If your child/children attend school physically, do you feel they are in danger?	No	109 (18.3%)	132 (9.6%)	<0.0001 *
	Neutral	339 (56.9%)	429 (31.2%)	
	Yes	148 (24.9%)	816 (59.3%)	
Parents’ concerns about the COVID-19 pandemic				
Currently, are you concerned and/or worried about the COVID-19 pandemic?	No	125 (21.0%)	595 (43.2%)	<0.0001 *
	Yes	471 (79.0%)	782 (56.8%)	
Parents’ perception of the COVID-19 vaccine				
Do you believe that children vaccination would help schools to return to face-to-face teaching?	No	100 (16.8%)	659 (47.9%)	<0.0001 *
	Yes	496 (83.2%)	718 (52.1%)	
Do you believe that children vaccination will protect the children from the COVID-19 infection?	No	80 (13.4%)	506 (36.8%)	<0.0001 *
	Yes	429 (72.0%)	485 (35.2%)	
	Not sure	87 (14.6%)	386 (28.0%)	
Parents’ awareness of the importance of the COVID-19 vaccine				
If the COVID-19 vaccine is available and affordable for children over 5 years old, would you give it for your children?	No	289 (48.5%)	664 (48.2%)	0.83
	Yes	307 (51.5%)	713 (51.8%)	
If your answer is “No”, how best describes the reason?	Side effects and safety of the vaccine	55 (9.2%)	118 (8.6%)	0.63
	The speed of making the vaccine	541 (90.8%)	1259 (91.4%)	

* significant result.

**Table 4:** Association between sociodemographic characteristics and parents’ perception of learning methods.

Variables		Learning Method			*p* Value
		Distance	Face-to-Face	Hybrid	
		*n* (%)			
Sex	Male (*n* = 251)	132 (52.6%)	67 (26.7%)	52 (20.7%)	0.058
	Female (*n* = 1722)	959 (55.7%)	349 (20.3%)	414 (24.0%)	
Age categories (years)	18–30 (*n* = 599)	288 (48.1%)	127 (21.2%)	184 (30.7%)	<0.0001
	31–40 (*n* = 579)	316 (54.6%)	149 (25.7%)	114 (19.7%)	
	41–50 (*n* = 571)	327 (57.3%)	104 (18.2%)	140 (24.5%)	
	51–60 (*n* = 224)	160 (71.4%)	36 (16.1%)	28 (12.5%)	
Residence area	Abu Dhabi (*n* = 863)	468 (54.2%)	157 (18.2%)	238 (27.6%)	<0.0001
	Dubai (*n* = 295)	131 (44.4%)	81 (27.5%)	83 (28.1%)	
	Northern Emirates (*n* = 815)	492 (60.4%)	178 (21.8%)	145 (17.8%)	
Educational level	High school (*n* = 12)	12 (100.0%)	0	0	<0.0001
	Undergraduate students (*n* = 317)	123 (38.8%)	69 (21.8%)	125 (39.4%)	
	Bachelor (*n* = 992)	548 (55.2%)	206 (20.8%)	238 (24.0%)	
	Master (*n* = 401)	270 (66.7%)	84 (20.7%)	51 (12.6%)	
	PhD (*n* = 247)	138 (55.9%)	57 (23.1%)	52 (21.1%)	
Educational background	Medical (*n* = 596)	208 (34.9%)	177 (29.7%)	211 (35.4%)	<0.0001
	Non-medical (*n* = 1377)	883 (64.1%)	239 (17.4%)	255 (18.5%)	
Living with an elderly person (65 years old and over)	No (*n* = 977)	553 (56.6%)	184 (18.8%)	240 (24.6%)	0.06
	Yes (*n* = 996)	538 (54.0%)	232 (23.3%)	226 (22.7%)	
If the COVID-19 vaccine is available and affordable for children over the age of five, would you give it to your child?	No 953 (48.4%)	620 (65.1%)	152 (15.9%)	181 (19.0%)	0.0001 *
	Yes 1017 (51.6%)	471 (46.3%)	264 (26.0%)	282 (27.7%)	

* significant result

**Table 5:** Multivariate logistic regression of the parents’ plans to return their children to face-to-face learning.

Variables	OR Exp(B)	95% C.I.		*p* Value
		Lower	Upper	
Sex				
Male	1.376	0.832	1.876	0.283
Female	Reference			
Age (years)				
18–30	1.847	1.165	2.926	0.009
31–40	2.142	1.407	3.262	<0.0001 *
41–50	1.456	0.946	2.242	0.088
>51	Reference			
Residence area				
Abu-Dhabi	0.930	0.720	1.202	0.580
Dubai	1.052	0.752	1.472	0.768
Northern Emirates	Reference			
Education level				
Undergraduate student	1.164	0.713	1.901	0.544
High school	-	-	-	-
Bachelor	0.835	0.581	1.199	0.328
Postgraduate (master’s degree)	0.695	0.465	1.037	0.075
Postgraduate (PhD)	Reference			
Educational background				
Non-medical	0.075	0.051	0.109	<0.0001 *
Medical background	Reference			
Hesitancy				
Parents said No to vaccine	0.423	0.386	0.461	<0.0001 *
Parents said YES	Reference			

* significant result

## Data Availability

Data will be available upon request.
